# Nutrition literacy: a key factor in public health improvement in India

**DOI:** 10.3389/fnut.2025.1643629

**Published:** 2025-09-30

**Authors:** Amar Mankar, Umesh Kawalkar, Zahiruddin Quazi Syed, Abhay Gaidhane

**Affiliations:** ^1^Department of Community Medicine, Datta Meghe Institute of Higher Education and Research, Wardha, India; ^2^Department of Community Medicine, Government Medical College, Akola, India; ^3^Datta Meghe Institute of Higher Education and Research, Wardha, India

**Keywords:** nutrition literacy, India, malnutrition, non-communicable disease, food literacy

## Abstract

Nutrition literacy is the capacity to obtain, understand, and apply nutrition information. It plays a pivotal role in addressing India’s coexistence of undernutrition, micronutrient deficiencies, and rising non-communicable diseases. This perspective synthesizes evidence on how nutrition, food, and health literacy shape dietary behavior and program uptake. This will highlight the rural–urban and adolescent–young adult gaps. We argue for participatory action research (PAR) approaches to co-design context-specific interventions and propose a multilevel strategy spanning schools, antenatal care, and community platforms. We present a conceptual framework linking literacy domains to improved dietary quality and NCD prevention, and outline priorities for measurement and policy integration in India.

## Introduction

India, a land of cultural diversity, is confronted with a multitude of nutritional challenges, ranging from malnutrition to the growing prevalence of diet-related diseases (1, 2). Undernutrition and micronutrient deficiencies remain common, especially in women and children. At the same time, lifestyle changes and urbanization have increased the number of people with obesity, diabetes, hypertension, and other non-communicable diseases (NCDs). This double burden affects health, productivity, and national development (3, 4).

Nutrition literacy means the ability to obtain, understand, and use nutrition information in daily life. It helps people choose balanced diets, read food labels, and avoid unhealthy food practices. Nutrition literacy is more than knowledge; it also includes skills and confidence to apply information for better health. When people have good nutrition literacy, they are more likely to improve their diet and reduce the risk of disease (5).

In India, nutrition literacy is especially important because of cultural diversity, socio-economic differences, and rapid dietary transitions. Rural and urban populations often face different challenges, and adolescents and young adults are particularly at risk of poor dietary habits (6, 7). Improving nutrition literacy can support existing programs such as POSHAN Abhiyaan, Mid-Day Meal Scheme, and Integrated Child Development Services (ICDS). This perspective highlights the importance of nutrition literacy, identifies gaps, and suggests practical strategies for public health action in India (8, 9).

A validated instrument for assessing nutrition literacy is essential for evaluating individuals’ comprehension of nutritional concepts in a standardized manner. Such an instrument facilitates the identification of knowledge gaps, monitors progress, and guides the development of effective educational strategies and interventions. These tools provide valuable insights for policymakers, assist healthcare providers in customizing their advice, and enable cross-cultural comparisons. Ultimately, they enhance public health planning and ensure that programs effectively improve dietary behaviors and health outcomes (10, 11).

Globally, validated instruments like the Food Literacy Assessment Tool (FLAT) and the Nutrition Literacy Assessment Instrument (NLAI) are available, yet their application in India remains limited (10, 12). While adapting these tools for India’s cultural and socio-economic diversity is crucial, there is an urgent need to develop a context-specific instrument for the Indian population. Such a tool would effectively capture local dietary patterns, traditional food practices, and the unique barriers to healthy eating. It could provide reliable evidence on nutrition literacy across various groups, including adolescents, women, and vulnerable communities, and inform the design of targeted interventions, program evaluations, and effective public health nutrition strategies in the country (13).

Designing such context-specific tools requires an understanding of India’s diverse dietary patterns, which are strongly shaped by culture, region, religion, and socio-economic factors. India’s rich cultural tapestry influences what people eat and how they make food choices (14). Dietary habits in India exhibit significant regional variation. For instance, in the coastal regions of Kerala and West Bengal, seafood constitutes a major component of the diet due to their proximity to the ocean, whereas in northern regions such as Punjab, wheat-based dishes like roti and paratha are dietary staples (15–17). Furthermore, religious practices exert a substantial influence on dietary choices; for example, vegetarianism is prevalent among Hindus, while Muslims frequently incorporate meat into their diets, particularly during religious festivals such as Eid (18). Nutrition literacy plays a crucial role in understanding and appreciating this diversity while promoting balanced diets within cultural contexts. By fostering awareness of traditional foods and culinary practices, nutrition literacy encourages individuals to make healthier food choices that align with their cultural preferences. Additionally, it aids in the preservation of indigenous knowledge related to food and nutrition, thereby contributing to efforts aimed at preserving cultural heritage (15, 19).

However, cultural diversity alone does not safeguard against poor nutrition. India continues to face a heavy burden of malnutrition, including persistent undernutrition, widespread micronutrient deficiencies, and a rising trend of overnutrition. These challenges underscore the urgent need to strengthen nutrition literacy as a core public health priority (20).

India continues to face a heavy burden of malnutrition, with anemia and protein–energy malnutrition remaining the most common public health concerns. According to National Family Health Survey-5 (NFHS-5, 2019–21) conducted by the Ministry of Health and Family Welfare, Government of India, anemia affects 67.1% of children under five and 57% of women of reproductive age. Iron deficiency is a major contributor to this high prevalence, leading to impaired cognitive development, reduced physical capacity, lower productivity, and increased risks of maternal and infant mortality (21). Protein–energy malnutrition is also widespread, with 35.5% of children under five stunted and 19.3% wasted, reflecting long-term risks for growth, learning, and overall health as per NFHS-5 (22). Micronutrient deficiencies further compound the problem. Vitamin A deficiency continues to affect about 62% of preschool children (WHO, 2009), increasing vulnerability to infections, weakening immunity, and raising the risk of blindness (23). Iodine deficiency persists as well, with 23% of households consuming inadequately iodized salt (NFHS-5, 2019–21), which is linked to poor cognitive development and thyroid-related disorders (24). The prevalence of inadequate absorbable zinc intake in India has risen from 17.1% in 1983 to 24.6% in 2011–12, affecting an additional 82 million people, and is projected to further increase with population aging and climate change, potentially placing 65 million more individuals at risk by 2050 (25). In addition, emerging deficiencies such as vitamin D and vitamin B12 are gaining attention. Vitamin D deficiency is increasingly reported, with links to bone health, immune dysfunction, and potential associations with chronic diseases, though nationwide prevalence data remain limited. Vitamin B12 deficiency varies across regions and dietary groups but is associated with neurological disorders, anemia, and cognitive decline, particularly among older adults (26).

India is currently experiencing a significant rise in overnutrition, overweight, and obesity, particularly in urban and peri-urban regions, alongside the persistent issue of undernutrition. According to NFHS-5, approximately one in four adults are classified as overweight or obese, with a higher prevalence observed among women and individuals from wealthier households. This nutritional transition, characterized by dietary shifts toward processed foods, sedentary lifestyles, and urbanization, is closely associated with the increasing incidence of diabetes, cardiovascular diseases, and other non-communicable diseases (NCDs). Consequently, addressing both undernutrition and overnutrition is imperative. Nutrition literacy emerges as a comprehensive strategy to address this dual burden by promoting healthier dietary choices and lifestyle behaviors (3, 27).

These nutritional challenges do not affect all population groups equally. Different stages of life present unique vulnerabilities and opportunities, making it essential to examine nutrition literacy across the life cycle from maternal and child health to adolescence, adulthood, and older age. Maternal nutrition literacy has a direct impact on pregnancy outcomes, infant feeding practices, and child growth. Women who are better informed about dietary diversity and supplementation are more likely to initiate breastfeeding early, adopt appropriate complementary feeding, and use available health services effectively (28, 29). The high levels of maternal anemia and child undernutrition reported in national surveys highlight the importance of equipping women with the skills to translate nutrition knowledge into practice. Evidence shows that interventions such as antenatal counseling, community health worker engagement, and mass media campaigns can significantly improve maternal diets and child feeding behaviors. Enhancing maternal literacy is therefore a critical step in breaking the intergenerational cycle of malnutrition (30). Adolescence is a critical window for establishing lifelong dietary patterns, yet poor nutrition literacy often results in unhealthy habits such as low intake of fruits and vegetables, reliance on processed foods, and irregular meal patterns (31, 32). These behaviors contribute not only to iron deficiency anemia but also to early onset of overweight and obesity, particularly in urban populations. Studies have shown that nutrition education delivered through schools, peer-led initiatives, and digital platforms can improve dietary choices and promote healthier behaviors. Investing in adolescent nutrition literacy is essential, as this group represents the future workforce and parents of the next generation, and their dietary practices will influence both current and long-term health outcomes (33).

Among adults, nutrition literacy plays a central role in preventing and managing non-communicable diseases (NCDs) (34). Individuals with stronger literacy skills are better able to interpret food labels, adhere to dietary guidelines, and make informed choices that reduce the risk of diabetes, hypertension, and cardiovascular disease. Poor literacy, on the other hand, contributes to unhealthy dietary patterns and delays in seeking care. In the elderly, nutrition literacy supports independence by guiding choices that prevent deficiencies of vitamin D, calcium, and vitamin B12, which are associated with frailty, bone disorders, and cognitive decline. Tailored interventions delivered through workplace programs, community centers, and healthcare providers can help adults and older populations improve diet quality, reduce disease complications, and enhance quality of life (35, 36).

Beyond individual health outcomes, nutrition literacy also plays a broader role at the community level, particularly in addressing issues of food security and equitable access to nutrition. Food security, ensuring all individuals have access to sufficient, safe, and nutritious food, remains a critical challenge in India. Despite progress, the country still grapples with issues of food availability, access, and utilization, particularly among vulnerable populations. According to the Food and Agriculture Organization (FAO), around 189.2 million people in India are undernourished, highlighting the persistent threat of hunger. Furthermore, malnutrition, both undernutrition and overnutrition, persists, with significant portions of the population experiencing deficiencies in essential nutrients (37–39). Nutrition literacy empowers communities to utilize available resources effectively, maximize the nutritional value of their diets, and advocate for policies that promote food security and healthy eating habits, thereby contributing to the overall well-being of the population.

Developing a validated nutrition literacy scale tailored to the Indian context is essential for accurate measurement and effective program design. Such a tool should capture local dietary practices, food environments, and cultural diversity, while undergoing rigorous testing for reliability and validity. Once developed, it can guide interventions, track progress, and inform policies to strengthen nutrition outcomes nationwide (40). Nutrition literacy, when measured and applied effectively, is instrumental in shaping public health interventions. Informed individuals are more likely to adopt healthy behaviors, participate in community-based programs, and benefit from school-, workplace-, and community-level initiatives. It also enhances the effectiveness of nutrition counseling and healthcare services by enabling individuals to make informed choices and take ownership of their health. At the policy level, nutrition literacy supports advocacy for healthy food environments, regulation of marketing practices, and multisectoral approaches to nutrition governance. Embedding nutrition literacy initiatives within existing systems, and aligning them with culturally relevant measurement tools, can strengthen program delivery, improve population health, and promote sustainable development in India (41–44).

Implementing these measurement and literacy initiatives in practice necessitates their integration with existing national nutrition programs and the adoption of innovative strategies like participatory action research (PAR). In India, efforts to combat micronutrient deficiencies are bolstered by targeted programs such as the National Iron Plus Initiative (NIPI), National Vitamin A Prophylaxis Program, and the National Iodine Deficiency Disorders Control Program (NIDDCP), alongside platforms like the Integrated Child Development Services (ICDS), Mid-Day Meal Scheme (MDMS), and National Nutrition Mission (POSHAN Abhiyaan). PAR can enhance these efforts by co-developing solutions with communities, frontline workers, and local institutions. The typical process involves: (i) community-led identification of literacy gaps and food environments; (ii) co-creation of messages, labels, and counseling aids in local languages; (iii) small-cycle testing (plan–do–study–act) within ICDS, school, and SHG platforms; and (iv) joint reflection using simple monitoring dashboards (literacy scores, dietary diversity, service uptake). Embedding PAR within district nutrition missions fosters local ownership and scalability. Despite these initiatives, gaps in nutrition literacy remain, impeding the effective implementation and uptake of micronutrient interventions (45–47). Bridging these gaps requires comprehensive strategies that integrate nutrition education, community engagement, and targeted interventions. A coordinated approach should encompass policy, delivery platforms, content, methods, and measurement, embedding literacy targets in POSHAN, school health, and NCD programs; utilizing schools, ANC/PNC, VHNDs, workplaces, and SHGs as delivery points; focusing on content such as label reading, budget-based meal planning, and myth-busting; employing methods like PAR-based co-design, peer educators, and digital micro-learning; and ensuring rigorous measurement through validated tools such as FLAT/NLAI with follow-up on dietary diversity, anemia services, and NCD risk behaviors (48, 49).

Nutrition literacy is emerging as a cornerstone for improving dietary behaviors, reducing the dual burden of malnutrition and non-communicable diseases, and strengthening public health in India. Despite multiple national programs and targeted initiatives, gaps in awareness, comprehension, and application of nutrition knowledge persist across the population. Developing and validating an India-specific nutrition literacy scale, embedding literacy goals into existing nutrition and health platforms, and employing participatory action research (PAR) can provide sustainable, culturally relevant solutions. By integrating nutrition literacy into schools, maternal and child health services, workplaces, and community structures, India can empower individuals to make healthier choices, enhance program uptake, and build community resilience. Strengthening nutrition literacy is not only a public health priority but also a pathway toward achieving broader goals of food security, social equity, and sustainable development.

## Data Availability

The original contributions presented in this study are included in this article/supplementary material, further inquiries can be directed to the corresponding authors.
